# Randomized, double-blind study comparing percutaneous electrolysis and dry needling for the management of temporomandibular myofascial pain

**DOI:** 10.4317/medoral.22488

**Published:** 2018-06-21

**Authors:** Ricardo Lopez-Martos, Luis-Miguel Gonzalez-Perez, Pablo Ruiz-Canela-Mendez, Francisco-Javier Urresti-Lopez, José-Luis Gutierrez-Perez, Pedro Infante-Cossio

**Affiliations:** 1 MD, PhD, Department of Oral and Maxillofacial Surgery, Virgen del Rocio University Hospital. Seville. Spain; 2 MD, DDS, PhD, Department of Oral and Maxillofacial Surgery, Virgen del Rocio University Hospital. Seville. Spain; 3 DPTSc, Department of Oral and Maxillofacial Surgery, Virgen del Rocio University Hospital. Seville. Spain

## Abstract

**Background:**

To assess whether the techniques of percutaneous needle electrolysis (PNE) and deep dry needling (DDN) used on trigger points (TrP) of lateral pterygoid muscle (LPM) can significantly reduce pain and improve function in patients with myofascial pain syndrome (MPS) compared to a control group treated with a sham needling procedure (SNP).

**Material and Methods:**

Sixty patients diagnosed with MPS in the LPM were selected and randomly assigned to one of three groups. The PNE group received electrolysis to the LPM via transcutaneous puncture. The DDN group received a deep puncture to the TrP without the introduction of any substance. In the SNP group, pressure was applied to the skin without penetration. Procedures were performed once per week for 3 consecutive weeks. Clinical evaluation was performed before treatment, and on days 28, 42 and 70 after treatment.

**Results:**

Statistically significant differences (*p*<0.01) were measured for the PNE and DDN groups with respect to pain reduction at rest, during chewing, and for maximum interincisal opening (MIO). Values for the PNE group showed significantly earlier improvement. Differences for PNE and DDN groups with respect to SNP group were significant (*p*<0.05) up to day 70. Evaluation of efficacy as reported by the patient and observer was better for PNE and DDN groups. No adverse events were observed for either of the techniques.

**Conclusions:**

PNE and DDN of the LPM showed greater pain reduction efficacy and improved MIO compared to SNP. Improvement was noted earlier in the PNE group than in the DDN group.

** Key words:**Myofascial pain syndrome, myofascial trigger points, percutaneous needle electrolysis, deep dry needling, lateral pterygoid muscle.

## Introduction

Myofascial pain syndrome (MPS) is a complex disorder of the musculoskeletal system, with multifactorial involvement, which has several clinical presentations in multiple areas of the body. One of these is the orofacial region, affecting the masticatory muscles and the temporomandibular joint (TMJ). MPS should be suspected in patients with pain and dysfunction of the masticatory muscles, together with the existence of trigger points (TrP) on palpation (1). TrPs are bands of muscle whose activation triggers tension and a deep and constant pain that can cause central excitation. The pain can be local or referred, and is characterized by its tendency to become chronic, limiting interincisal opening and causing muscle weakness as valid diagnostic criteria to differentiate myofascial temporomandibular disease from properly intra-articular disorders (1-3). It has been observed that the masseter and temporal muscles along with the lateral pterygoid muscle (LPM) are the muscles most frequently involved in active TrP in patients with temporomandibular disorders of myofascial origin (4).

In the temporomandibular area, TrPs associated with MPS usually do not resolve without treatment (4). Management can include the control of parafunctional habits, use of a mouth guard, and analgesic-anti-inflammatory therapy. This can be in conjunction with inactivation of TrPs by non-invasive methods, such as massages, ultrasound, muscle stretching with application of cold spray, and magnetic or electrical stimulation. Other mechanical treatments such as acupuncture or the direct application of medication to TrPs may be considered (5-7). To date, several minimally invasive methods have been described (8,9), with deep dry needling (DDN) being one of the techniques used to inactivate TrPs (1). Several studies in the literature have reported on its safety, efficacy and low cost in the management of MPS with LPM involvement (4,10,11).

Percutaneous needle electrolysis (PNE) consists in the application of a low intensity galvanic current through an acupuncture needle to accelerate tissue regeneration (12). It has been used successfully in various musculoskeletal pathologies, such as for the treatment of patellar tendinitis, tennis elbow, osteitis pubis and acute whiplash syndrome (13-16). However, to the best of our knowledge, no previous study has investigated the effect of PNE on TrPs of the masticatory muscles. The aim of the present study was to investigate if the PNE technique in LPM could reduce pain and improve mandibular mobility compared to DDN and a sham needling procedure (SNP). Secondary objectives were to assess the level of improvement in the general condition of the TMJ, as well as to assess the patient’s tolerance to the treatments performed and to side effects.

## Material and Methods

-Subjects:

A randomized, double-blind, single-centre clinical trial was carried out in the outpatient clinic of the Department of Oral and Maxillofacial Surgery of the Virgen del Rocío University Hospital, Seville (Spain), from June 2015 to June 2016.

The following diagnostic inclusion criteria were evaluated: a) age between 18 and 65 years, b) myogenic pain in the temporo-mandibular area of at least 6 months’ duration, c) moderately limited mandibular movement (interincisal opening limited to <40 mm and requiring passive stretching to increase opening by > 5 mm), according to Group I criteria of the RDC/TMD Consortium (17), and d) criteria satisfied for active TrPs in the LMP (pain upon intraoral palpation, limited range of movement, painful chin protrusion against resistance, lateralization of the contralateral side with mouth opening, and pain in the ipsilateral TMJ) according to the protocol used previously (1), following confirmation according to magnetic resonance study and panoramic radiography to rule out the presence of other conditions. Exclusion criteria were: a) the presence of TrPs in any other masticatory or cervical muscle, b) intra-articular pathology according to diagnostic criteria for temporo-mandibular disorders (17), c) dentofacial deformities, d) facial paralysis, e) vascular diseases, f) tension headache or migraine, g) previous infectious-inflammatory diseases of dental origin, h) belonephobia, i) fibromyalgia, j) depression or k) other medical comorbidities (diabetes, hypo- or hyperthyroidism).

The study was approved by the Hospital Ethics Committee (approval number 2014PI/083). All patients provided their informed consent prior to inclusion.

-Study design:

Patients were randomly assigned by Epidat 4.0 software to one of the three groups. The principal investigator and patients were all blinded to the assigned group until completion of the statistical analysis. The clinical evaluation of the patients was performed prior to treatment, and on days 28, 42 and 70 after treatment. Data was collected at each visit by the same observer.

The PNE group received a transcutaneous puncture in the LPM, according to the technique described by Koole *et al.* (18). Sterile stainless-steel needles (length 40 mm/ caliber 0.25 mm, with a cylindrical plastic guide, Agu-punt ®, Barcelona, Spain) were used for the muscle puncture. The puncture needles were connected to an electrosurgical device, and the electrotherapy equipment (EPI® Advanced Medicine, Barcelona, Spain) produced a continuous galvanic current of 2 mA for 3 seconds, three times through the cathode (electrosurgical scalpel), while the patient held the anode (hand electrode).

The puncture technique used for the DDN group was performed as previously described (2,11). A deep intramuscular puncture of the TPs was carried out without the introduction of any substance (dry puncture) (19). The objective was to provoke a jump reaction or local twitch response when the needle was inserted in a TrP (8). During the procedure, the operator used the volume of the electrotherapy equipment as a guide, simulating the EPI® technique. For the SNP group, the needle was pressed against the skin with its plastic protective tube, simulating a puncture, with the same noise reproduced with the EPI® equipment.

In all cases, the preauricular area was cleaned with alcohol 90% prior to the procedure, and the unilateral upper and lower bellies of the LPM were located manually intra- and extraorally. The procedures were performed once per week, for 3 consecutive weeks. Two weeks after each procedure, all subjects were instructed to perform concentric exercises with the masticatory muscles.

-Measures:

The main parameters evaluated were: a) pain at rest and with mastication according to a visual analogue scale (VAS), ranging from 0 (without pain) to 10 (worst pain imaginable), b) maximum interincisal opening (MIO) without causing pain or discomfort, using a jaw motion ruler to evaluate the distance between the upper and lower incisor in millimetres (Therabite® System ruler), and c) involvement of the TMJ, assessed by a 100-point questionnaire (0 worst case, 100 optimal) based on pain in daily activities (maximum 40 points), function (45 points) and mastication (15 points). Secondary efficacy results were the overall efficacy scores evaluated by the patients and the observer using a 5-point scale, ranging from 0 worst-possible outcome to the optimum outcome of 4. Tolerability to the treatment was evaluated by the patient and the observer using a 5-point scale, ranging from 0-very bad to 4-excellent. The type and frequency of adverse events were recorded at each visit.

-Statistical analysis:

Data were analysed with SPSS statistical software (IBM Statistics 19.0). Pre- and post-intervention comparisons of the variables in each group were performed with the Friedman test, while variations within each group were analysed with the Wilcoxon signed-rank test (with Bonferroni correction). Comparisons between the study groups were made with the Kruskal-Wallis test for each time point. If differences were detected between groups, the Mann-Whitney U test was used to detect where the difference was. Values of *p* <0.05 were considered to indicate statistical significance. When the Bonferroni correction was applied, the statistical significance was *p*<0.016.

## Results

Sixty patients were included in the study and randomly assigned to one of the three groups (20 patients in each group), from June 2015 to June 2016. The three groups had similar number of patients, and similar age distributions (median age of 39, range 18 to 62).  Table 1 shows the demographic characteristics and pain descriptions for the 60 participants. No significant differences were found between the 3 groups. Two patients from the DDN group and one patient from the SNP group dropped out of the trial. When performing the statistical analysis, the intention-to-treat analysis and the per-protocol analysis yielded identical results for all parameter measures; therefore, only the analysis per-protocol will be used to describe the results.

**Table 1:**
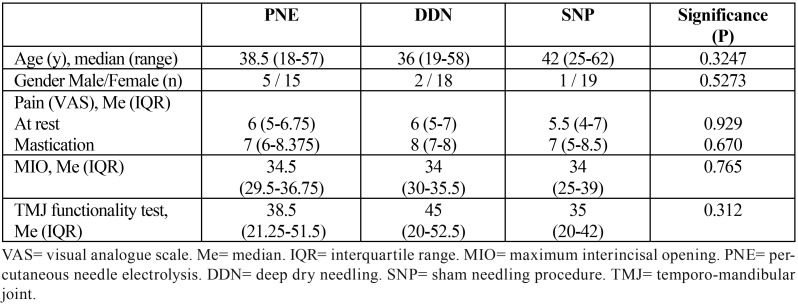
Demographic characteristics and pain description of all participants.

The reduction in pain at rest from day 0 to day 70 was statistically significant in the PNE and DDN groups (*p* <0.001) ( Table 2). In the PNE group, this difference was first evident on day 28 (*p*<0.0001), while in the DDN group it was significant at all time points (*p*= 0.004, *p*= 0.008 and *p*= 0.014). When comparing among the three groups, differences were statistically significant at all time-points in the study (*p*<0.001). Differences between the PNE and SNP groups were found for all three days in which data was collected (*p* = 0.002, *p* = 0.001 and *p* <0.001). Differences between the PNE and DDN groups were found between days 28 (*p* = 0.07) and 42 (*p* = 0.12), and between DDN and SNP at day 70 (*p* = 0.01). From day 0 to day 70, a significant reduction in pain with mastication was seen for the PNE and DDN groups (*p* <0.001) on day 28 (*p* = 0.001 and *p* <0.0001) ( Table 2). When comparing between the three groups, significant differences were seen at all time-points of the study (*p* = 0.016, *p* = 0.004 and *p* = 0.004). Differences between the PNE and SNP groups were significant at all time-points (*p* = 0.08, *p* = 0.05 and *p* = 0.02), while between the DDN and SNP groups differences were found on days 42 and 70 (*p* = 0.011 and *p* = 0.016).

**Table 2:**
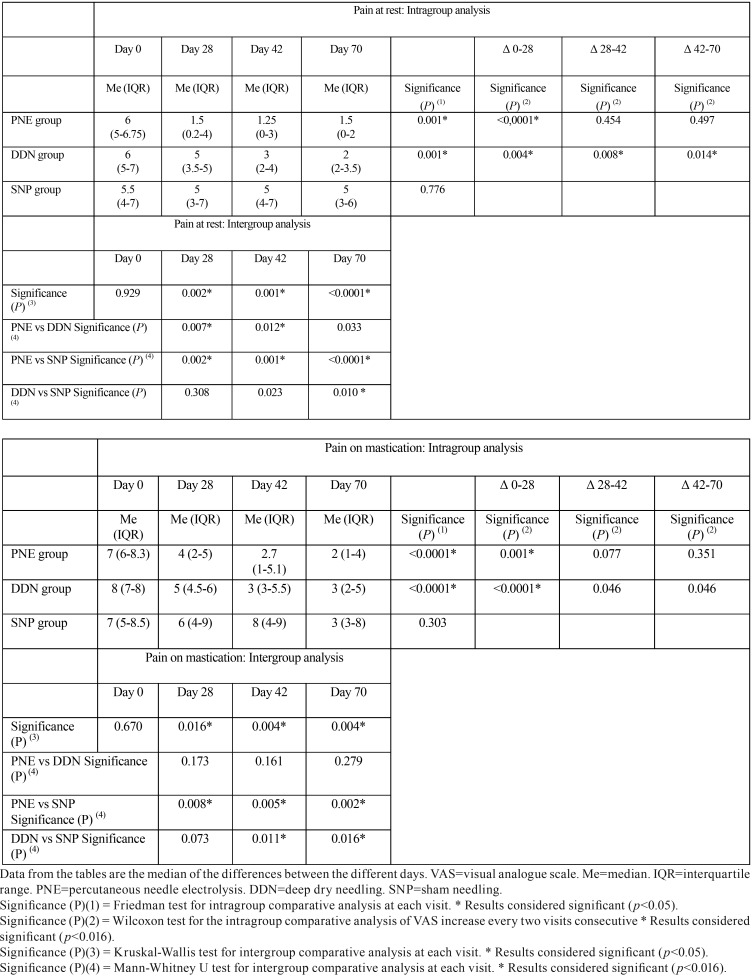
Pain at rest and pain on mastication, as measured on a 10-cm VAS.

MIO values improved significantly from day 0 to day 70 in both the PNE and DDN groups (*p* <0.001) ( Table 3), with a significant reduction also seen for both groups on day 28 (*p* <0.0001 and *p* = 0.001). When comparing between the three groups, differences were obtained on all three days of the study in which data was collected (*p* <0.001, *p* = 0.002 and *p* = 0.001). For the PNE group, the increase in MIO was higher than in both the DDN group (*p* = 0.001, *p* = 0.007 and *p* = 0.003) and the SNP group (*p* <0.001, *p* = 0.002 and *p* = 0.001) at all times.

**Table 3:**
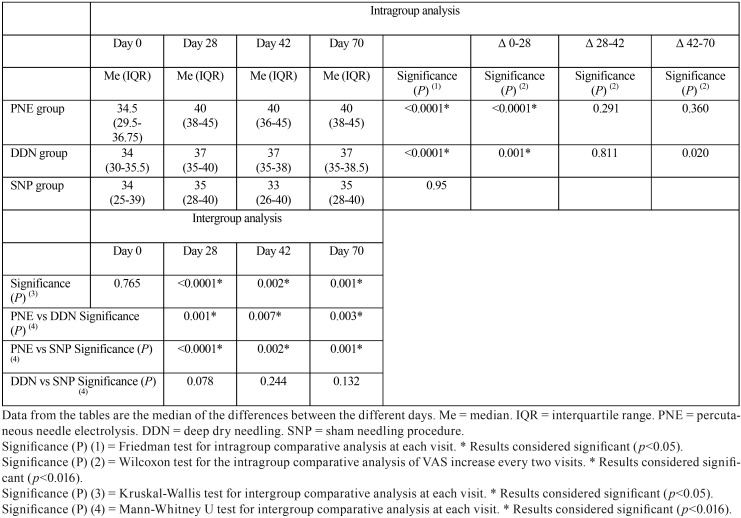
Maximal interincisal opening (MIO), as measured using a jaw motion ruler.

Values obtained in the 100-point questionnaire improved significantly between day 0 and day 70 in the three groups (*p* <0.001) ( Table 4). Significant differences were also found on day 28 for the PNE and DDN groups (*p* <0.0001 and *p* = 0.001). Again, when the three groups were compared, differences were significant on each of the three days in which data were recorded (*p* = 0.009, *p* = 0.004 and *p* <0.001). Values for the PNE group were higher than those for the SNP group on all three days (*p* = 0.006, 0 = 0.003 and *p* <0.001), and higher than the DDN group on day 70 (*p* = 0.001).

**Table 4:**
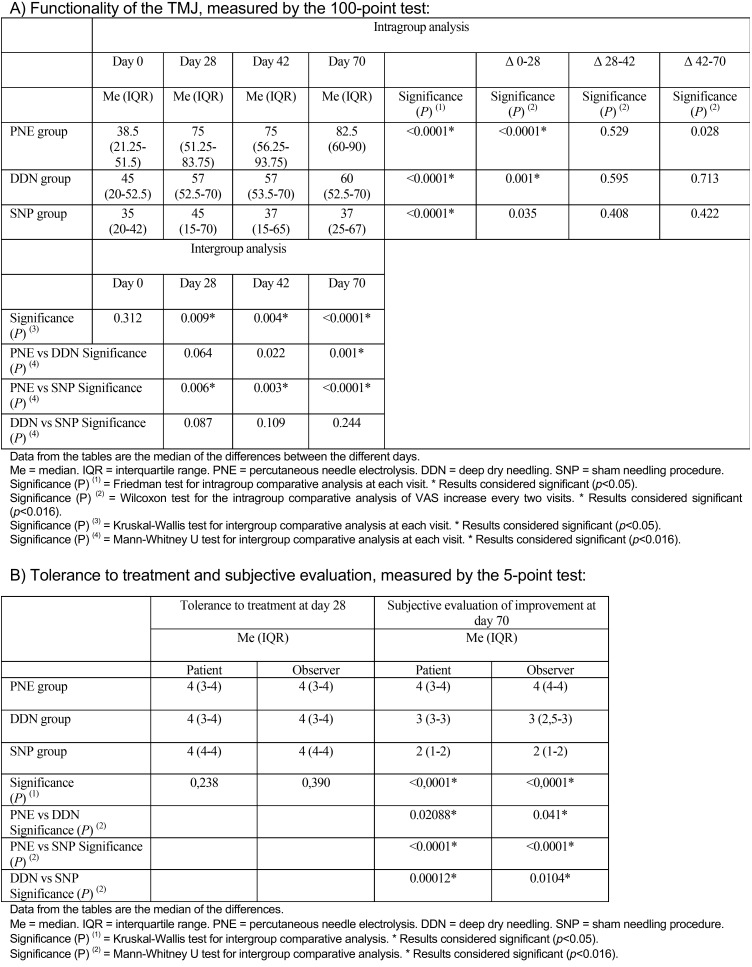
Functionality of the TMJ, measured by the 100-point test, and tolerance to treatment and subjective evaluation, measured by the 5-point test.

The only reported adverse effect was a self-limiting hematoma in one patient in the PNE group. No statistically significant differences in treatment tolerance were found between the three groups ( Table 4). The evaluation of the efficacy outcomes among the three groups was statistically significant both for the patient (*p* <0.0001) and the observer. When comparing between the three groups, this difference was greater for the PNE group than in the DDN and SNP groups, and in turn greater in the DDN group than in the SNP group, in terms of both patient and observer perception.

## Discussion

The objective of the present study was to evaluate the efficacy of PNE and DDN, two minimally-invasive techniques, applied three times to the LPM (once per week for three consecutive weeks). To do this, an analysis was made of the pain intensity at rest and with mastication, together with the measurements of the MIO ranges. The main findings can be outlined as follows: Compared to baseline values prior to treatment, PNE and DDN serve as effective treatments for MPS located in the LPM, improving pain, mandibular mobility and involvement of the TMJ (*p*<0.01). Both techniques immediate pain relief, and provided a stable outcome throughout the follow-up period as evidenced by significant improvements maintained until day 70 (*p* <0.05). It would seem that this effect was achieved earlier with PNE than with DDN. Pain reduction values were proportionately higher compared to those of improvements in MIO. And finally, when comparing these results with the SNP group, significant differences were generally obtained on all of the study days in which evaluations were made.

The therapeutic management of MPS should be based on a multidisciplinary approach where TrP inactivation is the fundamental objective. While various puncture methods have been described in the literature that attempt to inactivate myofascial TrPs (9,11), the principal difference between the different techniques consists of the injection, or not, of a substance into the TrP (dry puncture or wet puncture). No significant differences were reported in the literature between the use of DDN and the injection of any substance in the muscle belly (9).

DDN involves inactivation of TrPs via the insertion of an acupuncture needle without the administration of any substance. The mechanism of underlying the inactivation is not known, but the technique has been shown to provide effective pain relief and short-term functional recovery of muscles (20). The most accepted hypothesis of the technique’s mechanism of action is that the needle damages the motor endplate, which in turn causes denervation of the distal axon, and interruption of the central pain circuit (21). To ensure the success of the procedure, the local twitch response that occurs when the needle enters the TrP seems to be the best indicator to establish the diagnosis (8), although occasionally, identification of the local twitch response can be extremely difficult. The local twitch response corresponds to a spinal reflex with a momentary contraction of the fibers that make up the taut band of muscle. The patient describes it as a cramp or tingling at the time of the puncture.

A limited number of studies have investigated the use of DDN to treat TrP in the orofacial area. Fernandez-Carnero *et al.* (4) studied the use of DDN of the masseter muscle. Gonzalez-Perez *et al.* (11) compared DDN with analgesic medication for MPS by treating TrPs in the LPM, with pain relief achieved almost immediately in the DDN group. Recently, Blasco-Bonora & Martín-Pintado-Zugasti (3) used DDN on the temporal and masseter muscles. Taken together, these studies have reported statistically and clinically significant results in reducing both pain and dysfunction.

PNE is an emerging, minimally invasive physiotherapeutic technique that involves the application of direct current (galvanic) through a puncture needle such that used in DDN, which acts as a negative electrode and induces an electrochemical reaction in the area to which it is applied. Cell necrosis is caused by this reaction, which results in a local inflammatory process in the soft tissue, inducing phagocytosis and repair of the affected tissue (12). Tissue regeneration induced by PNE can restore function to the muscle, which is usually structurally damaged. PNE has been used to the present time to treat pathologies of the muscles and tendons, particularly in the lower limbs (13-16). To the best of our knowledge, no study has provided data on its use in orofacial pain as in our clinical trial. The paucity of other studies means that we are not available to compare our findings with others, making it difficult to arrive at definitive conclusions.

When comparing PNE with DDN in the present study, it was found that pain at rest and upon mastication decreased earlier in the PNE group. This was possibly because the technique combines both mechanical (needle) and electrical (galvanic current) stimulation (14). This effect could be explained by the inactivation of TrPs and by the acceleration of the regeneration of the damaged muscle with PNE. Three punctures were performed (one per week for three weeks) with application of a low intensity galvanic discharge in the LPM, with the aim of inactivating the TrPs. The slower improvement in the DDN group could have been due to the effect of the SNP and the blinding of the patients. In general, patients in the PNE group reported less post-puncture pain than in the DDN group. Improvements in MIO and in the 100-point test score were similar in the PNE and DDN groups, generating an improvement in the perceived quality of life of patients owing to the larger variety of foods they could eat.

The diagnosis of the presence of a TrP in the LPM is difficult due to its deep location. Painful intraoral palpation or limited mandibular opening are two common indirect clinical signs. The most reliable clinical test seems to be the painful protrusion against resistance (1,11,18,19). Exact localization of the TrP before the puncture can be achieved by palpation, ultrasound or electromyography, although their use is complex and not validated (22-25). In general, the precise puncture of the LPM is a simple, reliable and validated technique (11,18) achieved via a transcutaneous approach, with the two muscle bellies easily reached (26).

Tolerance to the treatment was the same in the three groups. The overall evaluation by the patients and observer of the effectiveness of the treatment, and the patients’ evaluation of treatment tolerance, were better for PNE and DDN than for SNP. Similarly, the overall efficacy evaluated both by patients and the observer was better for the PNE and DDN groups. No adverse reactions were detected with DDN, whereas in the PNE group a self-limiting hematoma was detected in one patient. As for any minimally invasive technique, both PNE and DDN were well tolerated without significant contraindications or costs (16). The strengths of the present trial lie in the fact that it was randomized, blinded and controlled, comparing two active interventions. Furthermore, data collection at standardized time-points during the postoperative period facilitated comparisons with the pre-operative baseline status.

This study has some limitations. Maintaining the blinding of patients in a clinical trial based on an intervention involving a muscle puncture is challenging. This type of effect makes it extremely difficult to conduct studies with a SNP in which participants are truly blinded. In the SNP group of this study, a superficial puncture of the skin was performed with the plastic protection applied (sham dry needling). In this way, the influence of the placebo effect of the procedure and / or the natural evolution of the TMD was controlled throughout the study. Tekin *et al.* (27) blinded participants by applying gentle pressure to the skin with the plastic protection; they described a mild effect in the first days after treatment, which was attributed to the stimulation of superficial cutaneous receptors. To achieve a true placebo effect, Mayoral *et al.* (28) conducted a study in which patients were placed under general anesthesia, and therefore had no way of knowing afterwards what procedure they had been subjected to. Another of the limitations identified here was the infrequently identified, exclusive affectation of the LPM, since disorders of the LPM usually co-exist with the involvement of other masticatory muscles such as the masseter or the temporal muscle (29). The evaluation was limited only to the effects observed in the short- and medium-term. To improve the validity of the study it would be important to increase the number of subjects, the time of follow-up, and the inclusion of patients in whom other masticatory muscles are affected. In addition, it would be interesting to assess the treatment in patients with fibromyalgia or depression (30), which in this study were excluded. The use of EMG in the diagnostic work-up, and as a treatment support for puncture of the LPM, could also be studied.

## Conclusions

In comparison with SNP, PNE and DDN of the LPM showed greater efficacy in relieving pain and improving MIO in patients with MPS in that muscle. The improvement was seen earlier in the PNE group than in the DDN group. No serious adverse events were observed with respect to any of the techniques used. Future studies should aim for greater validity by enrolling more patients and patients with other disorders of the temporo-mandibular region, to determine the true role of PNE in the management of MPS in the orofacial area.
